# Cerebral Pathophysiology in Extracorporeal Membrane Oxygenation: Pitfalls in Daily Clinical Management

**DOI:** 10.1155/2018/3237810

**Published:** 2018-03-18

**Authors:** Syed Omar Kazmi, Sanjeev Sivakumar, Dimitrios Karakitsos, Abdulrahman Alharthy, Christos Lazaridis

**Affiliations:** ^1^Division of Neurocritical Care and Vascular Neurology, Department of Neurology, Baylor College of Medicine, Houston, TX 77030, USA; ^2^Department of Neurology, Greenville Health System, University of South Carolina, Greenville, SC 29208, USA; ^3^Critical Care and Neurocritical Care Unit, King Saud Medical City, Riyadh 12746, Saudi Arabia; ^4^Department of Critical Care, Keck School of Medicine of USC, Los Angeles, CA 90033, USA

## Abstract

Extracorporeal membrane oxygenation (ECMO) is a life-saving technique that is widely being used in centers throughout the world. However, there is a paucity of literature surrounding the mechanisms affecting cerebral physiology while on ECMO. Studies have shown alterations in cerebral blood flow characteristics and subsequently autoregulation. Furthermore, the mechanical aspects of the ECMO circuit itself may affect cerebral circulation. The nature of these physiological/pathophysiological changes can lead to profound neurological complications. This review aims at describing the changes to normal cerebral autoregulation during ECMO, illustrating the various neuromonitoring tools available to assess markers of cerebral autoregulation, and finally discussing potential neurological complications that are associated with ECMO.

## 1. Introduction

The purpose of ECMO is to provide adequate oxygenated blood to the tissues by bypassing either the pulmonary or cardiopulmonary system in severe respiratory failure and/or cardiac failure, respectively. The ECMO circuit essentially consists of 4 components: (1) an inflow cannula which drains blood from the venous system, (2) a pump which provides flow in the circuit, (3) an oxygenator, which is responsible for oxygenating the venous blood, and (4) an outflow cannula which delivers the warmed oxygenated blood back into the venous or arterial system [1, 2]. In venovenous- (VV-) ECMO, the outflow cannula is directed into the venous system (typically the femoral, internal jugular, or subclavian vein), whereas in venoarterial- (VA-) ECMO the outflow cannula is inserted into the arterial system (usually the femoral artery but the subclavian, axillary, and common carotid arteries can be used as well) [3].

The effects on cerebral circulation for a patient on ECMO are complex and not precisely understood. This review aims at delineating the possible mechanisms of impaired cerebral autoregulation, identifying the different modalities to measure cerebral blood flow characteristics, and reviewing the neurological complications associated with ECMO.

## 2. Cerebral Autoregulation

**Figure 1:**
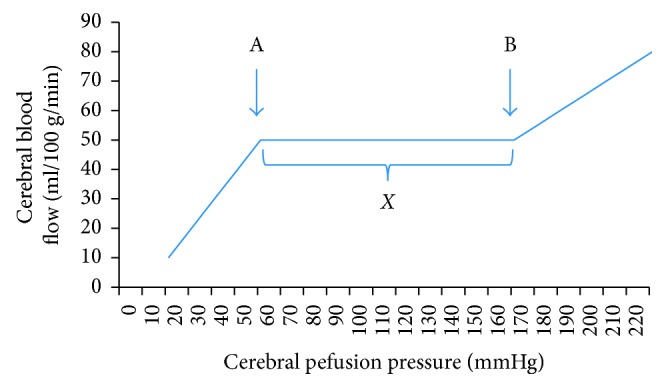
Lassen curve of autoregulation depicting variations of cerebral blood flow (CBF) over a range of cerebral perfusion pressures (CPP). Point A is the lower limit of the curve (LLA) after which a decrease in CPP will lead to reductions in CBF. Point B is the higher limit of the curve (HLA) after which an increase in CPP with increase CBF. The range of CPP depicted by *X* is the zone of autoregulation where the CBF remains constant over changes in CPP. This is regulated by vasoconstriction and vasodilation of cerebral arterioles.

Cerebral autoregulation is the ability of cerebral arterioles to maintain steady cerebral blood flow (CBF) over a varying range of mean arterial pressures (MAP) [4]. This is termed as cerebral pressure autoregulation and can be classically described using the Lassen curve [5], where MAP on the *x*-axis is plotted against CBF on the *y*-axis (Figure 1). A steady CBF is achieved by vasodilation and vasoconstriction of cerebral arterioles which in turn are influenced by neurogenic, myogenic, and metabolic mechanisms responding to changes in MAP [6]. These are complex processes and are poorly understood in the setting of different pathophysiological states. Neurogenic regulation is thought to be influenced via sympathetic and cholinergic pathways [7]. Myogenic regulation is carried out by the smooth muscle cells in the cerebral vessels which are responsible for myogenic tone and subsequently cerebral vascular resistance [8]. Metabolic regulation is related to changes in perineuronal concentrations of CO_2_, O_2_, K^+^, Ca^2+^, H^+^, and adenosine [9–13]. It should be kept in mind, however, that there is likely segmental and regional heterogeneity between the pial and parenchymal arteries and arterioles and their response to the above regulatory factors which can result in varying levels of CBF over the same range of CPP in different regions of the brain [8, 14–16].

In pathological conditions, cerebral autoregulation may become impaired. There may be focal impairment or global impairment depending on the pathological condition [17–19]. One of the most studied disease states resulting in impaired autoregulation is traumatic brain injury (TBI). Multiple studies have shown disturbed autoregulation after varying degrees of TBI even within “normal” ranges of CPP and CBF [20–23]. The loss of cerebral autoregulation can result in ischemia or edema, and hemorrhage even with slight changes in CPP. This is likely secondary to a combination of impaired neurogenic, myogenic, metabolic, and pressure dependent mechanisms [24]. Similar aberrations in cerebral autoregulation have been found in ischemic stroke [18, 25–27], intracerebral hemorrhage [28–30], and subarachnoid hemorrhage [31–33].

## 3. Cerebral Blood Flow Regulation on ECMO

Perhaps the greatest parallel that can be drawn to provide insight into autoregulation during ECMO is from the cardiopulmonary bypass (CPB) literature. On-pump CPB resembles VA-ECMO to a certain extent from which we can extrapolate similar changes in cerebral and systemic hemodynamics leading to changes in cerebral blood flow and autoregulation. In certain studies, up to 24% of patients have showed signs of impaired autoregulation during CPB, with numbers higher during the rewarming phase from hypothermia [34]. Cerebral blood flow was found to be arterial-pressure passive, resulting in a linear correlation of CBF and MAP indicating impaired cerebral autoregulation. Predictors of impaired autoregulation included male gender, average cerebral blood flow velocity, time-averaged cerebral oximetry index (COx) during CPB, PaCO_2_, and preoperative aspirin use according to one study which utilized near-infrared spectroscopy (NIRS) and transcranial Doppler (TCD) as tools to monitor cerebral autoregulation [35]; NIRS is a commonly used modality to monitor regional cerebral oxygen saturation during cardiac surgery, and its relationship with MAP can serve as an indicator of cerebral autoregulation. NIRS can provide information of cerebral oxygen supply and oxidative metabolic demand, from which a surrogate CBF can be derived [36–40]. TCD has been commonly used to measure CBF velocities from which CBF can be derived to give an estimation of CPP [41, 42]. In addition, TCD can also be used to detect microemboli [43]. The mean lower limit of autoregulation, after which a decrease in CPP results in a loss of CBF according to the Lassen curve, has been found to be 66 mmHg with values ranging from 40 to 90 mmHg in patients undergoing CPB [44]. Instead of targeting a specific number, it has been postulated that individualizing blood pressure management parameters using cerebral autoregulation monitoring can prevent neuronal injury [45].

Cerebral blood flow and autoregulation may be affected differently during ECMO. Initial animal studies suggested that CBF and oxygen metabolism did not change with the initiation of VA-ECMO [46]. However, at flow rates of less than 150 mL/kg/min, cerebral blood flow and oxygen delivery were found to decrease [47]. In addition, cerebral autoregulation was found to be impaired in newborn lambs on VA-ECMO at flow rates of 120–150 mL/kg/min [48]. These studies suggest that even though adequate cerebral blood flow can be maintained on VA-ECMO by adjusting flow rates, there are still aberrations in cerebral autoregulation. A possible explanation for these findings may be due to the pumps being used in the ECMO circuit. Previously used roller pumps have now been replaced by centrifugal pumps which provide continuous blood flow to the cerebral circulation. Pump flow is characterized by decreased systolic upstroke, lack of dichrotic notch, and continuous diastolic flow [49], and this loss of pulsatile flow may be responsible for the impairments seen in cerebral autoregulation. Indeed, low pulsatility indices have been demonstrated in patients undergoing ECMO along with decreased cerebral blood flow velocities [50, 51]. The pulsatility of CBF during partial bypass is likely related to preserved myocardial reserve while the regulation of CBF during prolonged bypass may be dependent on the presence of pulsatile flow [52].

Multiple pediatric studies have shown abnormal cerebral autoregulation in patients undergoing ECMO using noninvasive measures [53–55]. Most of these studies used the presence of a correlation between MAP and cerebral oxygen saturation using NIRS as a surrogate of cerebral autoregulation. A recent study correlated impaired cerebral autoregulation with abnormal neuroimaging findings [56]. This was a study of 25 pediatric patients who underwent either VA- or VV-ECMO on whom cerebral autoregulation was monitored using MAP and NIRS, focusing on a pressure-passive state. Brain imaging consisted of head ultrasound, CT scan of the head, and MRI of the brain post-ECMO. The study showed a higher degree of cerebral autoregulation impairment during ECMO, measured using wavelet transform coherence [57]; this impairment was associated with severe neuroimaging abnormalities. Another study of 6 pediatric patients showed a higher concordance between MAP and oxyhemoglobin concentrations with decreasing ECMO flow rates, indicative of a loss of autoregulation [50]. These studies suggest that the cerebral circulation undergoes some degree of impairment of autoregulation while on ECMO in the pediatric population, which may affect long-term neurological outcomes. Data to assess similar changes in adults are lacking.

There are various factors that can affect CBF in patients on ECMO. Out-flow cannulation site in VA-ECMO may contribute to variations in CBF. In peripheral VA-ECMO, the femoral artery is usually the site for out-flow cannulation in adults. The return of oxygenated blood directed towards the descending aorta via the femoral artery results in retrograde flow which can result in limb ischemia. In addition, retrograde blood flow creates additional afterload to the left ventricle (LV) which may lead to LV distension, reduced coronary flow, pulmonary edema, and hypoxemia [58]. The high flow states in VA-ECMO which serve to optimize systemic perfusion can compromise LV recovery by increasing afterload and hence pulmonary edema. It can be postulated that CBF may also be affected. In pediatric patients, the out-flow cannulation site can be the carotid artery, which would also potentiate alterations in CBF. Prior studies have shown that carotid artery ligation may produce an acute drop in CBF velocity at the onset of VA-ECMO [49, 59–63]. In addition, internal jugular vein occlusion due to in-flow cannulation can cause cerebral venous hypertension resulting in decreased CBF velocities [64]. A common clinical problem encountered in patients undergoing ECMO is dual circulation or Harlequin syndrome. This mainly represents upper body desaturation due to the position of the out-flow cannula in the distal aortic arch in cases of poor pulmonary function. Given that left ventricular unloading is often incomplete, the blood supply to the coronaries, brachiocephalic, and left carotid may not be adequately oxygenated due to being proximal to the out-flow cannula, resulting in lower oxygen saturations measured in the right arm. The risk of upper body desaturation can be minimized if the out-flow cannulation site is made in the ascending aorta (via sternotomy), axillary artery, subclavian artery, or the carotid artery [65–68]. However, positioning the in-flow cannula in the superior vena cava, instead of the femoral vein, has also been shown to improve upper body oxygenation while keeping the out-flow cannulation site the same [69]. This can reduce the risk of Harlequin syndrome and provide adequate oxygen delivery to the cerebral circulation.

Ventilatory management in ECMO patients can also affect the cerebral circulation. “Ultraprotective” mechanical ventilation (tidal volume of less than 4 mL/kg of ideal body weight) has been a favored strategy in ECMO patients given the reduced rates of pulmonary edema and lung injury [70, 71]; however, it has not been shown to reduce the number of ventilator free days [72]. Higher plateau pressures have been associated with increased mortality [73]. Early higher positive end expiratory pressures (PEEP) has been independently associated with improved overall mortality in patients on ECMO [74]. The effects of these ventilatory strategies on cerebral circulation are not completely understood. Increased PEEP has been shown to increase ICP and decrease CPP in brain injury patients; however, this has not been shown to be clinically significant [75].

Many centers are now employing the addition of an intra-arterial balloon pump (IABP) in conjunction with VA-ECMO for patients in cardiogenic shock due to evidence that it improves outcomes [76]. One study showed adequate carotid blood flow and oxygenation during cardiac arrest with the dual VA-ECMO and IABP regimen [77]. The addition of an IABP can influence CBF depending on the degree of native LV function. One study showed that the addition of an IABP in peripheral VA-ECMO significantly decreased CBF in myocardial stunning. However, as the LV recovered, the CBF tended to increase with the IABP [78]. Further studies need to be conducted to ascertain the changes in CBF and if these changes are clinically significant.

PaCO_2_ and pH are known to cause significant changes in CBF, and these parameters can rapidly change during ECMO [79]. In addition, peri-ECMO hemodynamic changes can affect the cerebral circulation. ECMO patients have severe derangements in their systemic hemodynamic status as baseline, and the addition of stress due to surgery, sedatives, paralytics, and vasopressors can induce a multitude of changes in the cerebral vasculature. If end-organ perfusion is not adequate, it can result in various systemic complications which may further affect CBF. It is difficult to ascertain exact individual etiologies and their effects on CBF during ECMO due to these reasons.

## 4. Neuromonitoring during ECMO

Neuromonitoring during ECMO is an important measure to obtain data on CBF features. A variety of noninvasive techniques have been suggested, each with their advantages and disadvantages, and these are employed across various institutions. The optimal neuromonitoring protocol has not been well established with current practices dependent on physician preference or device availability.

### 4.1. Near-Infrared Spectroscopy (NIRS)

NIRS is a noninvasive modality that is able to obtain a continuous measurement of cerebral oxygenation saturation usually by placing a frontal scalp electrode. The near-infrared light penetrates up to 2–2.5 cm into the brain and detects the concentrations of oxygenated and deoxygenated hemoglobin in the cerebral circulation [80–82]. This is usually expressed as a ratio of oxygenated hemoglobin to total hemoglobin termed regional cerebral oxygen saturation (rSO_2_). NIRS measures rSO_2_, which may be a reliable surrogate of CBF [83]. When rSO_2_ is plotted against a spectrum of MAPs, the cerebral oximetry index (COx) is generated which serves as a measure of cerebral autoregulatory vasoreactivity. When COx approaches 1, there is a strong correlation of MAP and rSO_2_ indicating a pressure-passive state of impaired autoregulatory vasoreactivity, and it has been validated in studies on adults [39, 84].

### 4.2. Transcranial Doppler

**Figure 2:**
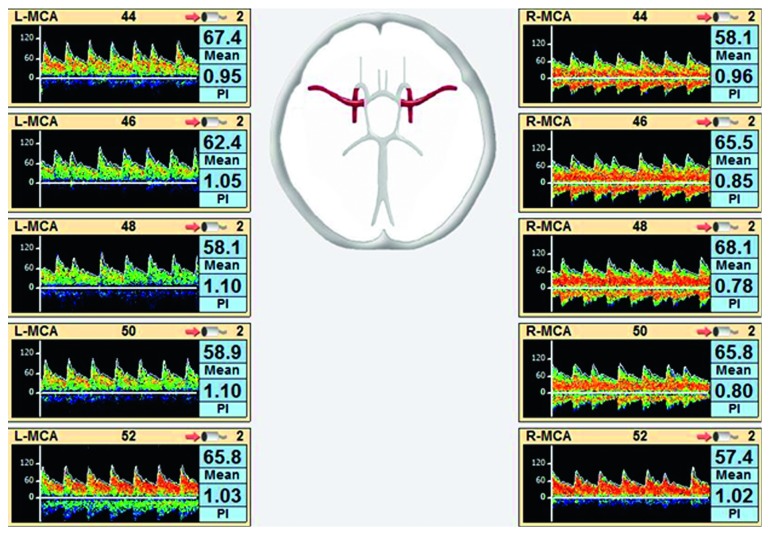
TCD waveforms of a patient on venovenous- (VV-) ECMO. Low mean cerebral blood flow velocities are observed in bilateral middle cerebral artery distributions with normal pulsatility indices. L-MCA: left middle cerebral artery; R-MCA: right middle cerebral artery; PI: pulsatility index.

**Figure 3:**
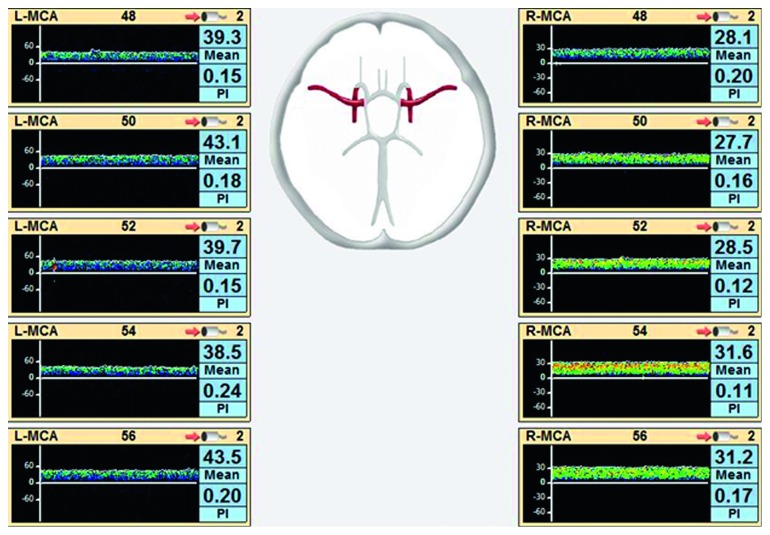
TCD waveforms of a patient on venoarterial- (VA-) ECMO. Low mean cerebral blood flow velocities are observed in bilateral middle cerebral artery distributions along with low pulsatility indices. L-MCA: left middle cerebral artery; R-MCA: right middle cerebral artery; PI: pulsatility index.

TCD has been used extensively in neurological and neurosurgical patients to monitor cerebral blood flow velocities. The device emits pulse wave ultrasounds that penetrate the brain and are reflected back after being scattered by moving red blood cells in the cerebral vasculature. The frequency is proportional to the blood flow velocity from which cerebral blood flow can be derived. The pulsatility index (PI) that is calculated as Doppler (systolic velocity – diastolic velocity)/mean velocity has been shown to be lower during ECMO initiation, and rising PI may be an indication of cerebral pathology [59, 62]. Furthermore, TCD can aid in detecting microemboli arising from the ECMO circuit in real time [85, 86]. The mean velocity index (Mx) is a derived variable that gives the strength of correlation between CBFV and CPP and has been described in the TBI literature [87]. Figures 2 and 3 are examples of TCD waveforms of patients on VV-ECMO and VA-ECMO, respectively (from our center).

### 4.3. Neuroimaging

Imaging of the brain during ECMO can be difficult; however, it is a helpful tool to aid in detecting neurological injury. In pediatric patients, head ultrasound can be used to detect acute neurological injury [88]. Computed tomography of the head, particularly if can be done portably, is a useful tool to rule out significant acute intracranial pathology. MRI of the brain cannot be used while on ECMO due to hardware incompatibility but is useful for evaluation of neurological injury post-ECMO. However, these imaging modalities give a snapshot of the brain architecture and do not give any information on dynamic cerebral hemodynamics.

### 4.4. Electroencephalogram

The electroencephalogram (EEG) monitors brain wave activity by placing electrodes on the scalp. When used continuously, it can provide important information regarding seizure activity; 50 to 80% of ECMO patients may have an abnormal EEG with electrographic seizures reported at 8–20% [89, 90]. However, cEEG is a scarce resource to have during the course of ECMO treatment; hence, many institutions may check periodic EEGs ranging from 20 min to 1 hour at a time.

### 4.5. Biomarkers

Various biomarkers of neuronal injury sampled from plasma have been used as markers of cerebral injury. Glial fibrillary acidic protein (GFAP), S100b, neuron specific enolase (NSE), intercellular adhesion molecule 5 (ICAM-5), brain-derived neurotrophic factor (BDNF), and monocyte chemoattractant protein 1/chemokine (c-c motif) ligand 2 (MCP-1/CCL-2) have been investigated in ECMO patients [91–94]. It is still not well established what the presence of these biomarkers signifies in terms of injury related to ECMO or from injury due to the initial diseased state. There is debate on the optimal cutoff values of these biomarkers as well as if serial assessment provides any meaningful information [95].

### 4.6. Other Neuromonitoring Techniques

Somatosensory evoked potentials (SSEP) provide information about cortical signals in the somatosensory cortex after a peripheral stimulus. This can be helpful to prognosticate cerebral injury if the cortical potentials are absent [96]. The optic nerve sheath diameter (ONSD) can be used at bedside to detect elevated ICP [97]. Diffuse correlation spectroscopy (DCS) is an emerging technique to noninvasively monitor regional CBF directly [98].

## 5. Neurological Complications on ECMO

Neurological injury causes significant morbidity and is a risk factor for mortality among critically ill patients undergoing ECMO. While the Extracorporeal Life Support Organization (ELSO) reports an overall survival rate of 55% with ECMO (http://www.elso.org), evidence of CNS infarction or hemorrhage confers a near fivefold increase in the odds for mortality, and poor survival rates of about 11% [99, 100]. Trends in prevalence of neurological complications from ECMO show that following an increase in prevalence rates between the early 1990s to early 2000s, there has been a significant decline in the prevalence of CNS injury in recent years [99]. Clinical seizures, ischemic strokes, and intracerebral hemorrhage are among the most common neurological complications reported.

### 5.1. Adults

Overall incidence of any clinical neurological event up to 19% has been reported with VA- and VV-ECMO [99, 101]. Reports from case series, population-based database, and the ELSO registry estimate a variable incidence rate for neurological complications among adults on ECMO: clinical seizures (1.8%–4%), cerebral infarction (2%–5.4%), and ICH (1.8%–19%) [99, 101–106]. Brain death is reported among 5%–21% of ECMO-treated adults [99, 100, 107–109]. A higher incidence of CNS complications is reported among patients on VA-ECMO when compared to VV-ECMO, based on data available from studies that separately analyzed patients based on ECMO-type [99, 101, 109–111]. An even higher prevalence of infarction and hemorrhage has been reported from postmortem neuropathological examinations of pediatric and adult ECMO nonsurvivors, showing that neurologic injury may be clinically undetected in 23–50% of cases [112–114].

Age, female sex, pre-ECMO cardiac arrest, use of inotropes, and post-ECMO hypoglycemia are factors shown to be independently associated with CNS complications with VA-ECMO [99, 102]. Rapid PaCO_2_ decrease at ECMO initiation and renal failure at ICU admission were independent predictors of ICH among patients undergoing VV-ECMO in one study [101]. In the same study, interestingly, disorders of hemostasis and anticoagulant use were not associated with neurological complications, including ICH.

### 5.2. Pediatrics

Neurological outcomes among pediatric patients undergoing ECMO have also been extensively studied. In a retrospective review of over 5000 pediatric patients aged 1 month to 18 years receiving ECMO from the ELSO database, the overall rate of acute severe neurological complications with ECMO was 13%, while patients undergoing ECMO-CPR had a higher incidence (26%) [115]. Other studies from the ELSO registry have found the rate of clinical seizures of up to 9.4% and 5.9%, ischemic stroke rates up to 7.4% and 4%, and ICH rates up to 7% and 6%, respectively, among neonates and children [116]. Risk factors for CNS complications in pediatrics include use of vasopressors, inotropes, serum bicarbonate administration, sepsis, severity of acidosis, pulmonary failure, elevated creatinine, and myocardial stunning [115].

### 5.3. Neurological Complications in ECMO-CPR

The incidence rates of neurological complications with ECMO-CPR are as high as 22% and are higher than with ECMO for other indications; in-hospital mortality rates can be as high as 89% among patients with neurological injury [99, 117]. Hypoxia, cardiac disease, acidosis, and need for CPR while on ECMO, presence of renal failure, cerebral hypoperfusion, and postresuscitation reperfusion injury are predictors of neurological injury after ECMO-CPR [108, 117, 118]. A cutoff value for arterial blood pH that can predict the occurrence of neurological complications has not been determined, and thus using blood pH as a sole predictor for decision-making in the context of neurological injury has been discouraged. It is possible that in many patients supported with ECMO-CPR, central nervous system (CNS) injury was sustained prior to ECMO deployment and as a consequence of cardiac arrest and shock.

### 5.4. Pathophysiology of Neurological Complications from ECMO

There is a lack of conclusive cause-effect relationship of CNS injury diagnosed during ECMO. The pathophysiology of neurological injury during ECMO is likely multifactorial and probably differs between VA-ECMO and VV-ECMO. While disorders from the ECMO circuit and oxygenator (hemolysis, thrombocytopenia, acquired Von Willebrand disease, and fibrinolysis) [119] are similar between VA- and VV-ECMO, pre-ECMO factors and the ECMO-induced metabolic changes could differ. Pre-ECMO illness severity and treatments (low blood pressure and low cerebral blood flow, acidosis, hypoxia, electrolyte disturbances, disorders of hemostasis secondary to hepatic failure from cardiogenic shock, to name a few), factors associated with ECMO implementation (reperfusion injury and embolic events from ECMO cannula), and post-ECMO events can contribute to CNS injury.

Loss of cerebral autoregulation during severe arterial hypertension or hypotension, hemorrhage secondary to anticoagulation, cerebral vasospasm, thromboembolism, and secondary brain injury from tissue edema surrounding an area of focal neurological injury are some mechanisms implicated in brain injury among ECMO patients [108–110, 114, 116, 120, 121]. A linear relationship between duration of ECMO and cerebral thromboembolic events was shown in one study [110]. Intracranial vascular hyperreactivity or hyporeactivity due to the loss of pulsatile blood flow during ECMO and in the presence of high dose vasoactive medications can cause tissue hypoperfusion and brain ischemia [50]. Long-lasting tissue hypoxia in the vascular distribution of the supraaortic blood vessels (Harlequin syndrome) [122] and suboptimal fluid and blood component management [121] further contribute to brain pathophysiology in an ECMO environment.

Factors specific to VV-ECMO include abrupt PaO_2_ and PaCO_2_ changes during initiation [123, 124]. Variations in arterial CO_2_ exert a profound influence on CBF. Around normal PaCO_2_, CBF changes by about 4% for each mmHg change in arterial PaCO_2_. Hypercapnia can cause cerebral vasodilation while hypocapnia causes constriction that can be marked. Cerebral vasodilation tends to increase cerebral blood volume and hence the intracranial pressure. Sudden changes in CO_2_ level (from hypercapnia to normocapnia or hypocapnia) during ECMO initiation can induce sudden decrement in CBF resulting in brain injury. A decrease in cerebral regional tissue oxygen saturation at VV-ECMO initiation linked to PaCO_2_ change has been demonstrated, which could be involved in pathogenesis of brain injury [125]. Avoiding rapid correction of hypercapnia by starting with a low sweep gas flow and gradually increasing with time is recommended to reduce the incidence of complications.

### 5.5. Healthcare Costs and Long-Term Neurological Outcomes

Neurological complications contribute significantly to the already-high healthcare costs associated with ECMO treatment. Hospitalization costs are more than US $100,000 higher among patients suffering from neurological complications of ECMO, than for patients without such complications [106]. Survival with good neurological outcomes has been estimated in the range of 13% to 65% among pediatric patients and up to 73% among adults undergoing ECMO and ECMO-CPR [126–128]. While there is scarcity of data on long-term neurological outcomes, one small study showed an unimpaired survival in nearly half of the adult survivors of ECMO during longer term (five years or more) neurological follow-up [129].

## 6. Practical Considerations

The management of ECMO can be challenging and complex. Most institutions have protocols to help guide optimal management; however, the neurological impact of ECMO is often over looked. At our institution (Baylor St. Luke's Medical Center, Houston, TX), we have instituted a neurosurveillance protocol termed the “Neuro-ECMO protocol.” This involves obtaining a CT head immediately after initiation of ECMO and a repeat scan 72 hours later. In addition, continuous electroencephalography is obtained and discontinued after 24 hours if there is no sign of seizures. Daily TCDs are also employed as well as NIRS, and daily neurological examinations are carried out by neurointensivists. We try to minimize sedation in order to obtain reliable neurological assessments when feasible. This can lead to the early detection of neurological complications. In addition, anticoagulation protocols mainly utilize heparin and take into account measuring partial thromboplastin times (PTT), measuring R time using thromboelastography (TEG), and obtaining anti-Xa and antithrombin 3 levels. PTT goals are usually set at 60 to 80 seconds if a patient has no risk factors. If two out of the three variables of PTT, R time and Anti Xa are therapeutic, the patient is considered to be adequately anticoagulated. Ventilator strategies aim for lung protective ventilation. A multidisciplinary approach is employed which involves cardiovascular anesthesiologists, cardiothoracic surgeons, cardiologists, perfusionists, and neurointensivists. We hope that the institution of such protocols and multidisciplinary teams can help improve the neurological impact associated with patients on ECMO.

## References

[1] Hill J. D., O’Brien T. G., Murray J. J. (1972). Prolonged extracorporeal oxygenation for acute post-traumatic respiratory failure (shock-lung syndrome). Use of the Bramson membrane lung. *New England Journal of Medicine*.

[2] Lequier L., Horton S. B., McMullan D. M., Bartlett R. H. (2013). Extracorporeal membrane oxygenation circuitry. *Pediatric Critical Care Medicine*.

[3] King C. S., Roy A., Ryan L., Singh R. (2017). Cardiac support: emphasis on venoarterial ECMO. *Critical Care Clinics*.

[4] Paulson O. B., Strandgaard S., Edvinsson L. (1990). Cerebral autoregulation. *Cerebrovascular and Brain Metabolism Reviews*.

[5] Lassen N. A. (1959). Cerebral blood flow and oxygen consumption in man. *Physiological Reviews*.

[6] Xiong L., Liu X., Shang T. (2017). Impaired cerebral autoregulation: measurement and application to stroke. *Journal of Neurology, Neurosurgery & Psychiatry*.

[7] Hamel E. (2006). Perivascular nerves and the regulation of cerebrovascular tone. *Journal of Applied Physiology*.

[8] Cipolla M. J., Li R., Vitullo L. (2004). Perivascular innervation of penetrating brain parenchymal arterioles. *Journal of Cardiovascular Pharmacology*.

[9] Lassen N. A., Christensen M. S. (1976). Physiology of cerebral blood flow. *British Journal of Anaesthesia*.

[10] Kuschinsky W., Wahl M. (1978). Local chemical and neurogenic regulation of cerebral vascular resistance. *Physiology Reviews*.

[11] Winn H. R., Welsh J. E., Rubio R. (1980). Brain adenosine production in rat during sustained alteration in systemic blood pressure. *American Journal of Physiology*.

[12] Kontos H. A. (1985). Oxygen radicals in cerebral vascular injury. *Circulation Research*.

[13] Wei E. P., Kontos H. A. (1988). Increased venous pressure causes myogenic constriction of cerebral arterioles during local hyperoxia. *Circulation Research*.

[14] Iadecola C. (2004). Neurovascular regulation in the normal brain and in Alzheimer’s disease. *Nature Reviews Neuroscience*.

[15] Edvinsson L., Owman C., Sjoberg N. O. (1976). Autonomic nerves, mast cells, and amine receptors in human brain vessels. A histochemical and pharmacological study. *Brain Research*.

[16] Faraci F. M., Mayhan W. G., Heistad D. D. (1987). Segmental vascular responses to acute hypertension in cerebrum and brain stem. *American Journal of Physiology-Heart and Circulatory Physiology*.

[17] Paulson O. B., Olesen J., Christensen M. S. (1972). Restoration of autoregulation of cerebral blood flow by hypocapnia. *Neurology*.

[18] Powers W. J., videen T. O., Diringer M. N. (2009). Autoregulation after ischaemic stroke. *Journal of Hypertension*.

[19] Georgiadis D., Schwarz S., evans D. H. (2002). Cerebral autoregulation under moderate hypothermia in patients with acute stroke. *Stroke*.

[20] Bouma G. J., Muizelaar J. P., Bandoh K., Marmarou A. (1992). Blood pressure and intracranial pressure-volume dynamics in severe head injury: relationship with cerebral blood flow. *Journal of Neurosurgery*.

[21] Czosnyka M., Smielewski P., Piechnik S., Steiner L. A., Pickard J. D. (2001). Cerebral autoregulation following head injury. *Journal of Neurosurgery*.

[22] Kirkness C. J., Mitchell P. H., Burr R. L., Newell D. W. (2001). Cerebral autoregulation and outcome in acute brain injury. *Biological Research For Nursing*.

[23] Lewelt W., Jenkins L. W., Miller J. D. (1980). Autoregulation of cerebral blood flow after experimental fluid percussion injury of the brain. *Journal of Neurosurgery*.

[24] Rangel-Castilla L., Gasco J., Nauta H. J., Okonkwo D. O., Robertson C. S. (2008). Cerebral pressure autoregulation in traumatic brain injury. *Neurosurgical Focus*.

[25] Tiecks F. P., Lam A. M., Aaslid R. (1995). Comparison of static and dynamic cerebral autoregulation measurements. *Stroke*.

[26] Novak V., Yang A. C., Lepicovsky L. (2004). Multimodal pressure-flow method to assess dynamics of cerebral autoregulation in stroke and hypertension. *BioMedical Engineering OnLine*.

[27] Panerai R. B., Jara J. L., Saeed N. P. (2015). Dynamic cerebral autoregulation following acute ischaemic stroke: comparison of transcranial Doppler and magnetic resonance imaging techniques. *Journal of Cerebral Blood Flow & Metabolism*.

[28] Oeinck M., Neunhoeffer F., Buttler K. J. (2013). Dynamic cerebral autoregulation in acute intracerebral hemorrhage. *Stroke*.

[29] Ma H., Guo Z. N., Liu J. (2016). Temporal course of dynamic cerebral autoregulation in patients with intracerebral hemorrhage. *Stroke*.

[30] Gould B., McCourt R., Asdaghi N. (2013). Autoregulation of cerebral blood flow is preserved in primary intracerebral hemorrhage. *Stroke*.

[31] Budohoski K. P., Czosnyka M., Smielewski P. (2012). Impairment of cerebral autoregulation predicts delayed cerebral ischemia after subarachnoid hemorrhage: a prospective observational study. *Stroke*.

[32] Otite F., Mink S., Tan C. O. (2014). Impaired cerebral autoregulation is associated with vasospasm and delayed cerebral ischemia in subarachnoid hemorrhage. *Stroke*.

[33] Jaeger M., Soehle M., Schuhmann M. U. (2012). Clinical significance of impaired cerebrovascular autoregulation after severe aneurysmal subarachnoid hemorrhage. *Stroke*.

[34] Joshi B., Brady K., Lee J. (2010). Impaired autoregulation of cerebral blood flow during rewarming from hypothermic cardiopulmonary bypass and its potential association with stroke. *Anesthesia & Analgesia*.

[35] Ono M., Joshi B., Brady K. (2012). Risks for impaired cerebral autoregulation during cardiopulmonary bypass and postoperative stroke. *British Journal of Anaesthesia*.

[36] Czonsnyka M., Brady K., Reinhard M., Smielewski P., Steiner L. (2009). Monitoring of cerebrovascular autoregulation: facts, myths, and missing links. *Neurocritical Care*.

[37] Edmonds H. L. (2006). Pro: all cardiac surgical patients should have intraoperative cerebral oxygenation monitoring. *Journal of Cardiothoracic and Vascular Anesthesia*.

[38] Brady K., Lee J., Kibler K. (2007). Continuous time-domain analysis of cerebrovascular autoregulation using near-infrared spectroscopy. *Stroke*.

[39] Brady K., Joshi B., Zweifel C. (2010). Real time continuous monitoring of cerebral blood flow autoregulation using near-infrared spectroscopy in patients undergoing cardiopulmonary bypass. *Stroke*.

[40] Smielewski P., Kirkpatrick P., Minhas P., Pickard J. D., Czosnyka M. (1995). Can cerebrovascular reactivity be measured with near-infrared spectroscopy?. *Stroke*.

[41] Polito A., Ricci Z., Chiara L. (2006). Cerebral blood flow during cardiopulmonary bypass in pediatric cardiac surgery: the role of transcranial Doppler-a systematic review of the literature. *Cardiovascular Ultrasound*.

[42] Kosir G., Tetickovic E. (2011). Intraoperative transcranial Doppler ultrasonography monitoring of cerebral blood flow during coronary artery bypass grafting. *Acta Clinica Croatica*.

[43] O’Brien J. J., Butterworth J., Hammon J. W., Morris K. J., Phipps J. M., Stump D. A. (1997). Cerebral emboli during cardiac surgery in children. *Anesthesiology*.

[44] Joshi B., Ono M., Brown C. (2012). Predicting the limits of cerebral autoregulation during cardiopulmonary bypass. *Anesthesia & Analgesia*.

[45] Hori D., Ono M., Rappold T. E. (2015). Hypotension after cardiac operations based on autoregulation monitoring leads to brain cellular injury. *Annals of Thoracic Surgery*.

[46] Short B. L., Walker L. K., Gleason C. A., Jones M. D., Traystman R. J. (1990). Effect of extracorporeal membrane oxygenation on cerebral blood flow and cerebral oxygen metabolism in newborn sheep. *Pediatric Research*.

[47] Rosenberg A. A., Kinsella J. P. (1992). Effect of extracorporeal membrane oxygenation on cerebral hemodynamics in newborn lambs. *Critical Care Medicine*.

[48] Short B. L., Walker L. K., Bender K. S., Traystman R. J. (1993). Impairment of cerebral autoregulation during extracorporeal membrane oxygenation in newborn lambs. *Pediatric Research*.

[49] Raju T. N., Kim S. Y., Meller J. L. (1989). Circle of Willis blood velocity and flow direction after common carotid artery ligation for neonatal extracorporeal membrane oxygenation. *Pediatrics*.

[50] O’Brien N. F., Hall M. W. (2013). Extracorporeal membrane oxygenation and cerebral blood flow velocity in children. *Pediatric Critical Care Medicine*.

[51] Kavi T., Esch M., Rinsky B., Rosengart A., Lahiri S., Lyden P. D. (2016). Transcranial Doppler changes in patients treated with extracorporeal membrane oxygenation. *Journal of Stroke and Cerebrovascular Diseases*.

[52] Taylor G. A., Martin G. R., Short B. L. (1989). Cardiac determinants of cerebral blood flow during extracorporeal membrane oxygenation. *Investigative Radiology*.

[53] Papademetriou M. D., Tachtsidis I., Elliot M. J., Hoskote A., Elwell C. E. (2012). Multichannel near infrared spectroscopy indicates regional variations in cerebral autoregulation in infants supported on extracorporeal membrane oxygenation. *Journal of Biomedical Optics*.

[54] Ingyinn M., Rais-Bahrami K., Viswanathan M., Short B. L. (2006). Altered cerebrovascular responses after exposure to venoarterial extracorporeal membrane oxygenation: role of the nitric oxide pathway. *Pediatric Critical Care Medicine*.

[55] Caicedo A., De Smet D., Naulaers G. (2011). Cerebral tissue oxygenation and regional oxygen saturation can be used to study cerebral autoregulation in prematurely born infants. *Pediatric Research*.

[56] Tian F., Morriss M. C., Chalak L. (2017). Impairment of cerebral autoregulation in pediatric extracorporeal membrane oxygenation associated with neuroimaging abnormalities. *Neurophotonics*.

[57] Tian F., Tarumi T., Liu H., Zhang R., Chalak L. (2016). Wavelet coherence analysis of dynamic cerebral autoregulation in neonatal hypoxic–ischemic encephalopathy. *NeuroImage: Clinical*.

[58] Pavlushkov E., Berman M., Valchanov K. (2017). Cannulation techniques for extracorporeal life support. *Annals of Translational Medicine*.

[59] Taylor G., Catena L., Garin D. (1987). Intracranial flow patterns in infants undergoing extracorporeal membrane oxygenation: preliminary observations with Doppler US. *Radiology*.

[60] Taylor G., Short B., Glass P. (1988). Cerebral hemodynamics in infants undergoing extracorporeal membrane oxygenation: further observations. *Radiology*.

[61] Matsumoto J., Babcock D., Brody A., Weiss R. G., Ryckman F. G., Hiyama D. (1990). Right common carotid artery ligation for extracorporeal membrane oxygenation: cerebral blood flow velocity measurement with Doppler duplex US. *Radiology*.

[62] Van de Bor M., Walther F., Gangitano E., Snyder J. R. (1990). Extracorporeal membrane oxygenation and cerebral blood flow velocity in new-born infants. *Critical Care Medicine*.

[63] Lohrer R., Bejar R., Simko A. (1992). Internal carotid artery blood flow velocities before, during and after extracorporeal membrane oxygenation. *American Journal of Diseases of Children*.

[64] Weber T. R., Kountzman B. (1996). The effects of venous occlusion on cerebral blood flow characteristics during ECMO. *Journal of Pediatric Surgery*.

[65] Javidfar J., Brodie D., Costa J. (2012). Subclavian artery cannulation for venoarterial extracorporeal membrane oxygenation. *ASAIO Journal*.

[66] Schachner T., Nagiller J., Zimmer A., Laufer G., Bonatti J. (2005). Technical problems and complications of axillary artery cannulation. *European Journal of Cardio-Thoracic Surgery*.

[67] Maclaren G., Butt W., Best D., Donath S., Taylor A. (2007). Extracorporeal membrane oxygenation for refractory septic shock in children: one institution’s experience. *Pediatric Critical Care Medicine*.

[68] Rollins M. D., Hubbard A., Zabrocki L., Barnhart D. C., Bratton S. L. (2012). Extracorporeal membrane oxygenation cannulation trends for pediatric respiratory failure and central nervous system injury. *Journal of Pediatric Surgery*.

[69] Lindfors M., Frenckner B., Sartipy U., Bjällmark A., Broomé M. (2017). Venous cannula positioning in arterial deoxygenation during veno-arterial extracorporeal membrane oxygenation—a simulation study and case report. *Artificial Organs*.

[70] Terragni P. P., Del Sorbo L., Mascia L. (2009). Tidal volume lower than 6 ml/kg enhances lung protection: role of extracorporeal carbon dioxide removal. *Anesthesiology*.

[71] Frank J. A., Gutierrez J. A., Jones K. D., Allen L., Dobbs L., Matthay M. A. (2002). Low tidal volume reduces epithelial and endothelial injury in acid-injured rat lungs. *American Journal of Respiratory and Critical Care Medicine*.

[72] Bein T., Weber-Carstens S., Goldmann A. (2013). Lower tidal volume strategy (≈3 ml/kg) combined with extracorporeal CO_2_ removal versus ‘conventional’ protective ventilation (6 ml/kg) in severe ARDS: the prospective randomized Xtravent-study. *Intensive Care Medicine*.

[73] Schmidt M., Zogheib E., Rozé H. (2013). The PRESERVE mortality risk score and analysis of long-term outcomes after extracorporeal membrane oxygenation for severe acute respiratory distress syndrome. *Intensive Care Medicine*.

[74] Schmidt M., Stewart C., Bailey M. (2015). Mechanical ventilation management during extracorporeal membrane oxygenation for acute respiratory distress syndrome: a retrospective international multicenter study. *Critical Care Medicine*.

[75] Boone M. D., Jinadasa S. P., Mueller A. (2017). The effect of positive end-expiratory pressure on intracranial pressure and cerebral hemodynamics. *Neurocritical Care*.

[76] Doll N., Kiaii B., Borger M. (2004). Five-year results of 219 consecutive patients treated with extracorporeal membrane oxygenation for refractory postoperative cardiogenic shock. *Annals of Thoracic Surgery*.

[77] Bělohlávek J., Mlček M., Huptych M. (2012). Coronary versus carotid blood flow and coronary perfusion pressure in a pig model of prolonged cardiac arrest treated by different modes of venoarterial ECMO and intraaortic balloon counterpulsation. *Critical Care*.

[78] Yang F., Jia Z.-s., Xing J.-l. (2014). Effects of intra-aortic balloon pump on cerebral blood flow during peripheral venoarterial extracorporeal membrane oxygenation support. *Journal of Translational Medicine*.

[79] Meng L., Gelb A. W. (2015). Regulation of cerebral autoregulation by carbon dioxide. *Anesthesiology*.

[80] Ferrari M., Quaresima V. (2012). A brief review on the history of human functional near-infrared spectroscopy (fNIRS) development and fields of application. *Neuroimage*.

[81] Cui X., Bray S., Reiss A. L. (2010). Functional near infrared spectroscopy (NIRS) signal improvement based on negative correlation between oxygenated and deoxygenated hemoglobin dynamics. *Neuroimage*.

[82] Dehghani H., White B. R., Zeff B. W., Tizzard A., Culver J. P. (2009). Depth sensitivity and image reconstruction analysis of dense imaging arrays for mapping brain function with diffuse optical tomography. *Applied Optics*.

[83] Moerman A., De Hert S. (2017). Recent advances in cerebral oximetry. Assessment of cerebral autoregulation with near-infrared spectroscopy: myth or reality?. *F1000Research*.

[84] Steiner L. A., Pfister D., Strebel S. P. (2009). Near-infrared spectroscopy can monitor dynamic cerebral autoregulation in adults. *Neurocritical Care*.

[85] Zanatta P., Forti A., Bosco E. (2010). Microembolic signals and strategy to prevent gas embolism during extracorporeal membrane oxygenation. *Journal of Cardiothoracic Surgery*.

[86] Marinoni M., Migliaccio M., Trapani S. (2016). Cerebral microemboli detected by transcranial Doppler in patients treated with extracorporeal membrane oxygenation. *Acta Anaesthesiologica Scandinavica*.

[87] Zeiler F. A., Donnelly J., Menon D. K. (2017). Continuous autoregulatory indices derived from multi-modal monitoring: each one is not like the other. *Journal of Neurotrauma*.

[88] Bembea M. M., Felling R., Anton B., Salorio C. F., Johnston M. V. (2015). Neuromonitoring during extracorporeal membrane oxygenation: a systematic review of the literature. *Pediatric Critical Care Medicine*.

[89] Streletz J., Bej M. D., Graziani L. J. (1992). Utility of serial EEGs in neonates during extracorporeal membrane oxygenation. *Pediatric Neurology*.

[90] Gannon C. M., Kornhauser M., Gross G. W. (2001). When combined, early bedside head ultrasound and electroencephalography predict abnormal computerized tomography or magnetic resonance brain images obtained after extracorporeal membrane oxygenation treatment. *Journal of Perinatology*.

[91] Nguyen D. N., Huyghens L., Wellens F., Schiettecatte J., Smitz J., Vincent J. L. (2014). Serum s100B protein could help to detect cerebral complications associated with extracorporeal membrane oxygenation (ECMO). *Neurocritical Care*.

[92] Bembea M. M., Rizkalla N., Freedy J. (2015). Plasma biomarkers of brain injury as diagnostic tools and outcome predictors after extracorporeal membrane oxygenation. *Critical Care Medicine*.

[93] Floerchinger B., Philipp A., Foltan M. (2014). Neuron-specific enolase serum levels predict severe neuronal injury after extracorporeal life support in resuscitation. *European Journal of Cardio-Thoracic Surgery*.

[94] Gazzolo D., Abella R., Marinoni E. (2009). New markers of neonatal neurology. *Journal of Maternal-Fetal & Neonatal Medicine*.

[95] Lorusso R., Taccone F. S., Belliato M. (2017). Euro-ELSO working group on neurologic monitoring and outcome. brain monitoring in adult and pediatric ECMO patients: the importance of early and late assessments. *Minerva Anestesiologica*.

[96] Zanatta P., Linassi F., Mazzarolo A. P. (2015). Pain-related somatosensory evoked potentials: a potential new tool to improve the prognostic prediction of coma after cardiac arrest. *Critical Care*.

[97] Raffiz M., Abdullah J. M. (2017). Optic nerve sheath diameter measurement: a means of detecting raised intracranial pressure in adult traumatic and non-traumatic neurosurgical patients. *American Journal of Emergency Medicine*.

[98] Durduranand T., Yodh A. G. (2014). Diffuse correlation spectroscopy for non-invasive, microvascular cerebral blood flow measurement. *NeuroImage*.

[99] Lorusso R., Barili F., Mauro M. D. (2016). In-hospital neurologic complications in adult patients undergoing venoarterial extracorporeal membrane oxygenation: results from the Extracorporeal Life Support Organization Registry. *Critical Care Medicine*.

[100] Brogan T. V., Thiagarajan R. R., Rycus P. T., Bartlett R. H., Bratton S. L. (2009). Extracorporeal membrane oxygenation in adults with severe respiratory failure: a multi-center database. *Intensive Care Medicine*.

[101] Luyt C. E., Bréchot N., Demondion P. (2016). Brain injury during venovenous extracorporeal membrane oxygenation. *Intensive Care Medicine*.

[102] Kasirajan V., Smedira N. G., McCarthy J. F., Casselman F., Boparai N., McCarthy P. M. (1999). Risk factors for intracranial hemorrhage in adults on extracorporeal membrane oxygenation. *European Journal of Cardio-Thoracic Surgery*.

[103] Noah M. A., Peek G. J., Finney S. J. (2011). Referral to an extracorporeal membrane oxygenation center and mortality among patients with severe 2009 influenza A(H1N1). *JAMA*.

[104] Australia and New Zealand Extracorporeal Membrane Oxygenation (ANZ ECMO) Influenza Investigators, Davies A., Jones D. (2009). Extracorporeal Membrane Oxygenation for 2009 Influenza A(H1N1) Acute Respiratory Distress Syndrome. *JAMA*.

[105] Hemmila M. R., Rowe S. A., Boules T. N. (2004). Extracorporeal life support for severe acute respiratory distress syndrome in adults. *Annals of Surgery*.

[106] Nasr D. M., Rabinstein A. A. (2015). Neurologic complications of extracorporeal membrane oxygenation. *Journal of Clinical Neurology*.

[107] Lan C., Tsai P. R., Chen Y. S., Ko W. J. (2010). Prognostic factors for adult patients receiving extracorporeal membrane oxygenation as mechanical circulatory support–a 14-year experience at a medical center. *Artificial Organs*.

[108] Thiagarajan R. R., Brogan T. V., Scheurer M. A., Laussen P. C., Rycus P. T., Bratton S. L. (2009). Extracorporeal membrane oxygenation to support cardiopulmonary resuscitation in adults. *Annals of Thoracic Surgery*.

[109] Ko W. J., Lin C. Y., Chen R. J., Wang S. S., Lin F. Y., Chen Y. S. (2002). Extracorporeal membrane oxygenation support for adult postcardiotomy cardiogenic shock. *Annals of Thoracic Surgery*.

[110] Rastan A. J., Dege A., Mohr M. (2010). Early and late outcomes of 517 consecutive adult patients treated with extracorporeal membrane oxygenation for refractory postcardiotomy cardiogenic shock. *Journal of Thoracic and Cardiovascular Surgery*.

[111] Wu M. Y., Lin P. J., Lee M. Y. (2010). Using extracorporeal life support to resuscitate adult postcardiotomy cardiogenic shock: treatment strategies and predictors of short-term and midterm survival. *Resuscitation*.

[112] Rastan A. J., Lachmann N., Walther T. (2006). Autopsy findings in patients on postcardiotomy extracorporeal membrane oxygenation (ECMO). *International Journal of Artificial Organs*.

[113] Reed R. C., Rutledge J. C. (2010). Laboratory and clinical predictors of thrombosis and hemorrhage in 29 pediatric extracorporeal membrane oxygenation nonsurvivors. *Pediatric and Developmental Pathology*.

[114] Mateen F. J., Muralidharan R., Shinohara R. T., Parisi J. E., Schears G. J., Wijdicks E. F. (2011). Neurological injury in adults treated with extracorporeal membrane oxygenation. *Archives of Neurology*.

[115] Cengiz P., Seidel K., Rycus P. T., Brogan T. V., Roberts J. S. (2005). Central nervous system complications during pediatric extracorporeal life support: incidence and risk factors. *Critical Care Medicine*.

[116] Hervey-Jumper S. L., Annich G. M., Yancon A. R., Garton H. J., Muraszko K. M., Maher C. O. (2011). Neurological complications of extracorporeal membrane oxygenation in children. *Journal of Neurosurgery: Pediatrics*.

[117] Barrett C. S., Bratton S. L., Salvin J. W., Laussen P. C., Rycus P. T., Thiagarajan R. R. (2009). Neurological injury after extracorporeal membrane oxygenation use to aid pediatric cardiopulmonary resuscitation. *Pediatric Critical Care Medicine*.

[118] Cheng R., Hachamovitch R., Kittleson M. (2014). Complications of extracorporeal membrane oxygenation for treatment of cardiogenic shock and cardiac arrest: a meta-analysis of 1,866 adult patients. *Annals of Thoracic Surgery*.

[119] Heilmann C., Geisen U., Beyersdorf F. (2012). Acquired von Willebrand syndrome in patients with extracorporeal life support (ECLS). *Intensive Care Medicine*.

[120] Polito A., Barrett C. S., Wypij D. (2013). Neurologic complications in neonates supported with extracorporeal membrane oxygenation. An analysis of ELSO registry data. *Intensive Care Medicine*.

[121] Mehta A., Ibsen L. M. (2013). Neurologic complications and neurodevelopmental outcome with extracorporeal life support. *World Journal of Critical Care Medicine*.

[122] Wong J. K., Smith T. N., Pitcher H. T., Hirose H., Cavarocchi N. C. (2012). Cerebral and lower limb near-infrared spectroscopy in adults on extracorporeal membrane oxygenation. *Artificial Organs*.

[123] Schmidt M., Tachon G., Devilliers C. (2013). Blood oxygenation and decarboxylation determinants during venovenous ECMO for respiratory failure in adults. *Intensive Care Medicine*.

[124] Schmidt M., Pellegrino V., Combes A., Scheinkestel C., Cooper D. J., Hodgson C. (2014). Mechanical ventilation during extracorporeal membrane oxygenation. *Critical Care*.

[125] Muellenbach R. M., Kilgenstein C., Kranke P. (2014). Effects of venovenous extracorporeal membrane oxygenation on cerebral oxygenation in hypercapnic ARDS. *Perfusion*.

[126] Morris M. C., Wernovsky G., Nadkarni V. M. (2004). Survival outcomes after extracorporeal cardiopulmonary resuscitation instituted during active chest compressions following refractory in-hospital pediatric cardiac arrest. *Pediatric Critical Care Medicine*.

[127] Nadkarni V. M., Larkin G. L., Peberdy M. A. (2006). National Registry of Cardiopulmonary Resuscitation Investigators. First documented rhythm and clinical outcome from in-hospital cardiac arrest among children and adults. *JAMA*.

[128] Meaney P. A., Nadkarni V. M., Cook E. F. (2006). American Heart Association National Registry of Cardiopulmonary Resuscitation Investigators. Higher survival rates among younger patients after pediatric intensive care unit cardiac arrests. *Pediatrics*.

[129] Risnes I., Wagner K., Nome T. (2006). Cerebral outcome in adult patients treated with extracorporeal membrane oxygenation. *Annals of Thoracic Surgery*.

